# Molecular Targets of Natural Products for Chondroprotection in Destructive Joint Diseases

**DOI:** 10.3390/ijms21144931

**Published:** 2020-07-13

**Authors:** Thanasekaran Jayakumar, Periyakali Saravana Bhavan, Joen-Rong Sheu

**Affiliations:** 1 Graduate Institute of Medical Sciences, College of Medicine, Taipei Medical University, Taipei 110, Taiwan; tjaya_2002@yahoo.co.in; 2 Department of Zoology, Bharathiar University, Coimbatore 641046, Tamil Nadu, India; bhavan@buc.edu.in; 3 Department of Pharmacology, School of Medicine, College of Medicine, Taipei Medical University, Taipei 110, Taiwan

**Keywords:** arthritis, MMPs, natural products, chondroprotection, signaling pathways

## Abstract

Osteoarthritis (OA) is the most common type of arthritis that occurs in an aged population. It affects any joints in the body and degenerates the articular cartilage and the subchondral bone. Despite the pathophysiology of OA being different, cartilage resorption is still a symbol of osteoarthritis. Matrix metalloproteinases (MMPs) are important proteolytic enzymes that degrade extra-cellular matrix proteins (ECM) in the body. MMPs contribute to the turnover of cartilage and its break down; their levels have increased in the joint tissues of OA patients. Application of chondroprotective drugs neutralize the activities of MMPs. Natural products derived from herbs and plants developed as traditional medicine have been paid attention to, due to their potential biological effects. The therapeutic value of natural products in OA has increased in reputation due to their clinical impact and insignificant side effects. Several MMPs inhibitor have been used as therapeutic drugs, for a long time. Recently, different types of compounds were reviewed for their biological activities. In this review, we summarize numerous natural products for the development of MMPs inhibitors in arthritic diseases and describe the major signaling targets that were involved for the treatments of these destructive joint diseases.

## 1. Introduction

Osteoarthritis (OA) is the most common type of joint disease that affect millions of people worldwide and it primarily cause disability in the aged population, affecting about 80% of individuals over the age of 75 [1]. Increased damage of cartilage degradation is the hallmark of this destructive joint disease. In the cartilage matrix, proteoglycan and collagen exist as major elements, and damage of proteoglycan could induce cartilage degeneration [2], followed by the catabolism of collagen fibrils, which increases the loss of cartilage structural integrity [3]. Matrix metalloproteinases (MMPs)-induced cartilage degeneration is controlled by endogenous tissue inhibitors of metalloproteinase (TIMPs) [4], and the disproportion in the ratio of TIMPs and MMPs could lead to a persistent matrix destruction in OA [5]. It was proposed that MMPs inhibition should be considered a therapeutic strategy in preventing cartilage degradation, which occurs in the arthritic process [6]. Although several inhibitors of the MMPs have been proposed as important therapeutic agents, there is a lack of evidence related to the inhibition of MMPs by natural compounds for chondroprotection in the destructive joint diseases.

## 2. MMPs and Osteoarthritis

Matrix metalloproteinases are proteolytic enzymes that restore and degrade extracellular matrix (ECM) proteins and their components. MMPs enzymes break down cartilages and their levels are elevated in joint tissues of patients with rheumatoid arthritis (RA) and OA [7]. Joint inflammation and joint degenerative diseases are associated with increased level of MMPs; so far, 23 MMP proteins have identified in humans [8]. Chondrocytes are vital cells that exist in the cartilage and are mostly accountable for affecting ECM in joint space. Chondrocytes synthesize collagen type II and aggrecan, which are similar to ECM and secrete proteolytic MMPs.

Collagenases, such as MMP-1 and 13 are highly degraded collagens in the cartilage and bone. In osteoarthritis, the components of cartilage matrix are hydrolyzed quickly and results in cartilage degradation. Collagenase-1 (MMP1) exists in various cells, including chondrocytes [9]. MMP13 (collagenase-3) majorly induce collagen degrading activity, especially of type II collagen [10], and this enzyme plays a major role in the degradation of cartilage. Various MMP inhibitors were established and verified for potential clinical use [11]. Moreover, MMP-13 expression co-express with CII degradation in OA lesions, indicating that this enzyme exerts a pivotal role in cartilage degradation in OA [12]. Moreover, immunohistochemistry has revealed the presence of MMP13-specific type II collagen degradation products and MMP13 enzymes in OA cartilage [13,14]. Though MMPs1, 8, and 13 have reported the only mammalian enzymes that degrade native fibrillary collagen types I, II, and III, other MMPs, including MMP2 and MMP14 also possess this activity [15,16]. The collagenases enzyme MMP2 and MMP9 degrade type IV collagen, gelatin, and elastin, which are complicated in joint diseases [17].

## 3. Inflammatory Cytokines in Osteoarthritic Chondrocytes

Inflammatory cytokines are the most important class of compounds contributing to the pathogenesis of OA. In the cartilages, interleukin-1β (IL-1) and tumor necrosis factor-α (TNF) were shown to induce of MMPs 1, 3, 9, and 13 expression [18], and these cytokines are found to be a suitable model in the human SW1353 chondrosarcoma cell line that is compatible with primary chondrocytes in OA [19]. Interleukin-1β stimulates the release of degenerative MMPs enzymes from chondrocytes and synoviocytes, and extracellular matrix proteins in chondrocytes [20]. IL-1β is also involved in the osteoclastogenesis and bone resorption, which is augmented in rheumatoid arthritis (RA) joints [21]. Apoptotic chondrocyte death in articular cartilage was observed in clinical specimens from RA and OA cartilages [22]. A previous study reported that anti-TNF-α treatment with a TNF antibody, gives a continued reduction of pain symptoms in OA [23], therefore, antagonists to TNF-α might serve as a potential beneficial strategy to decrease OA pain in patients [24].

Interleukin (IL)-1 is one of the most essential degrading cytokines secreted by chondrocytes in arthritic joint disease [25]. Augmented levels of IL-1 were noticed in synovial fluids from RA and OA patients [26], and its over expression in osteoarthritic cartilage tissue was also reported by Teslow et al. [13]. A high level of IL-1 receptor type 16 was observed in osteoarthritic chondrocytes, compared to normal chondrocytes, and inhibitors of IL-1 converting enzyme, a protease crucial for IL-1β processing, was found to reduce collagen-induced arthritis [27]. Moreover, opposing data were noted in a potential up- and down-regulation of IL-1β, in osteoarthritic cartilage [28]. IL-1 was reported to produce excessive effects in chondrocytes, including (i) a major reduction in the expression of collagen type II [29]; (ii) over expression of MMP-1, 3, and 13 [30]; and (iii) solid stimulation of intercellular mediators like leukemia inhibitory factor and IL-6. Interleukin-6, another well-recognized cytokine involved in cartilage degradation, was reported to connect with hyperalgesia and hypersensitivity in joint tissues [31]. This cytokine played a vital role in the progression of RA, as its level was found to increase in the serum and synovial fluid of arthritic patients [32]. Interleukin-6 reacted remarkably to primary afferent neurons [33], and hence it could play a role in pain transmission in arthritic states. In the skeleton system, IL-6 triggers osteoclasts and stimulates the synovium to produce MMPs that are responsible for degrading cartilage in OA [34]. Therefore, inhibiting IL-6 over-expression in synovial fibroblasts (SF) is believed to be an auspicious method to prevent OA progression, in which the clarification of molecular mechanisms underlying IL-6 over-expression in SF is essential.

## 4. Phorbol 12-myristate 13-acetate (PMA) in Osteoarthritis

Phorbol 12-myristate 13-acetate are the tetracyclic diterpenoids commonly identified for their tumor-promoting activity. PMA imitate the action of diacyl glycerol (DAG), an inducer of protein kinase C (PKC), which regulates several signal transduction pathways and other cellular metabolic activities. The release of histamine, cytokines, proteases, and the activation of NADPH oxidase, are highly associated with PMA [35], and lead to the induction of inflammation. Proteases and PMA are prospective agents for starting acute inflammation. Phorbol 12-myristate 13-acetate was reported to involve MMPs overexpression in activated human chondrocytes [19]. A study showed that polymorphonuclear neutrophil on the surface of cartilage was able to cause substantial breakdown of cartilage proteoglycan when they were activated by PMA [36]. Our previous study found that PMA induced MMP1 and 13 expressions in SW1353 chondrocyte cells [37], which were reduced by the treatment of sesamol.

## 5. Animal Models for Osteoarthritis

Osteoarthritis in animal models are well recognized, as they are proposed to be an essential and alternate tool for studies of OA in humans [38,39]. Animal models can provide the resources of studying the disease progression of OA, and support in the growth of therapeutic drugs and biological markers for identifying the disease [38]. The monosodium iodoacetate (MIA) model was extensively used to test for the potential analgesic agents in pain-associated studies, as this model was easily reproducible and mimicked with the histoarchitecture of human OA [40]. Studies proposed that the damages of MIA-induced OA are concentration- and time-dependent [40,41]. To explore the nociceptive mechanisms in the pathology of OA, several studies with MIA animal models were performed. The intraarticular injection of MIA into the knee joint of rats is the most generally used experimental model for inducing OA. Monosodium iodoacetate inhibits the activity of glyceraldehyde-3-phosphate dehydrogenase of articular chondrocytes, leading to a distraction of glycolytic energy metabolism and synthetic routes, and ultimately inducing cell death [42]. Hence, a continuous damage of the articular cartilage and abrasions in the subchondral bone could be observed in this model, which strictly resembled that in OA patients [42]. A study established that several MMP inhibitors [43] revered the upregulation of MMPs in knee cartilage from the MIA-injected rats. 

### Dosage of MIA in Osteoarthritis Model

The quantity of MIA injection is an essential factor for the development of OA and it was evidenced by observing the grade of histopathological alterations [40]. It was reported that 0.2 mg of MIA is considered as the typical low dose, whereas 1.0 mg is considered as high dose in the MIA model [44]. Additionally, a previous study found dose- and time-dependent MIA-induced arthritis [44]. Another study reported 1.0 mg of MIA to be the highest and most effective dose for inducing OA in rats [45]. In our earlier study, an intraarticular injection of 25 μL (10 mg/mL concentration of MIA) of MIA into the left knee was carried out for 7 days, to induce OA [37].

## 6. Role of Nuclear Factor Kappa-B (NF-κB) in Osteoarthritis

Nuclear Factor κB is a major transcription factor that was identified in numerous organisms, from flies to mammals [46]. Heterodimer of p50 and p65 (Rel-A) proteins is the most extensively circulated kB-binding factor in mammals. Nuclear Factor κB contributes a central role in host defense for various reactions via the rapid induction of gene expression. The expression of several inflammatory cytokines, and adhesion molecules involved in tumor metastasis was regulated by NF-κB. Nuclear Factor κB and its related genes were found to be dysregulated in various pathological conditions, including toxic/septic shock, atherosclerosis, and cancer [47,48]. Unlike other transcription factors, NF-κB family proteins occur in cytoplasm in an inactive state through its inhibitory subunit, called IκBα. IκB phosphorylation and subsequent degradation lets NF-κB translocate to the nucleus. Many agents, such as cytokines, mitogens, lipopolysaccharides (LPS), protein synthesis inhibitors, H_2_O_2_, UV light, and PMA [49], exacerbate this stimulation.

In OA, the phenotype of articular chondrocytes could be transformed by a chain of cellular actions [50]. In chondrocytes, NF-κB facilitates the inflammatory responses in OA that could lead to ECM damage and cartilage destruction [51]. Though hundreds of genes that are involved in inflammation, differentiation, and apoptosis were found to be regulated by NF-κB, these processes were also controlled by post-translational modifications, such as the reversible acetylation of p65. More than five acetylation sites were identified within p65, and alteration of these sites controlled the DNA-binding and transcriptional activities of NF-κB [52,53,54]. Activation of NF-κB was highly linked between p65 acetylation and deacetylation, and therefore, NF-κB inhibition via deacetylation was a possible therapy for OA. The application of a new therapeutic strategy would increase the understanding of the involvement of NF-κB in OA. Compounds or drugs that can downregulate the activation of NF-κB were considered to be potentially beneficial for intervention. A previous study of ours demonstrated that seasamol, derived from the sesame seed diminished MMPs-1, 9, and 13 expression in TNF-α-, IL-1β-, or PMA-stimulated chondrocytes via the inhibition of NF-κB activation, such as inhibition of p65 nuclear translocation and IκB-α degradation [37]. Another study explored that treatment of chondrocytes with curcumin inhibited IL-1β-induced NF-κB activation, via the suppression of IκB-α phosphorylation, IκB-α degradation, p65 phosphorylation, and its nuclear translocation [55]. 

## 7. Molecular Targets of Natural Products and Their Therapeutic Intervention for Osteoarthritis

The molecular mechanisms responsible for osteoarthritis development are tightly intricate and not well described. In articular cartilage, a balance between progressive and destructive mechanisms sustains extracellular matrix homeostasis and moves toward destructive processes that are related to OA. Chondrocyte cells synthesize extracellular matrix components, including collagens, proteoglycans, and non-collagen proteins. Biochemical features and mechanical packing are supposed to play significant roles in disease development, though chondrocyte interacts with molecular signals that fluctuate in altered regions and at different phases of the disease. Here, various chondroprotective natural compounds and their molecular targets are summarized, as shown in Table 1.

The current treatment strategies for OA are fully involved in decreasing symptoms, the recovery function, and delay time to surgery. There are three kinds of therapeutic agents, disease-modifying OA drugs (DMOADs), such as hyaluronic and glucosamine; nonsteroidal anti-inflammatory drugs (NSAIDs) such as loxoprofen and nabumetone; and steroid and biological response modifiers, which are all clinically proven to dismiss the severity of OA [56]. However, these drugs only have worthy effects on OA, but there were reports that chronic use could induce side effects on gastrointestinal tract [57]. A study showed that piascledine, a combination of the non-saponifiable components of avocado and soybean oils, holds gifted anti-inflammatory arthritis symptoms [58]; some researchers also studied small anti-inflammatory molecules from the natural sources for developing novel treatment strategy [59], but the scientific validation of their anti-arthritic value is still inadequate. 

**Figure 1:**
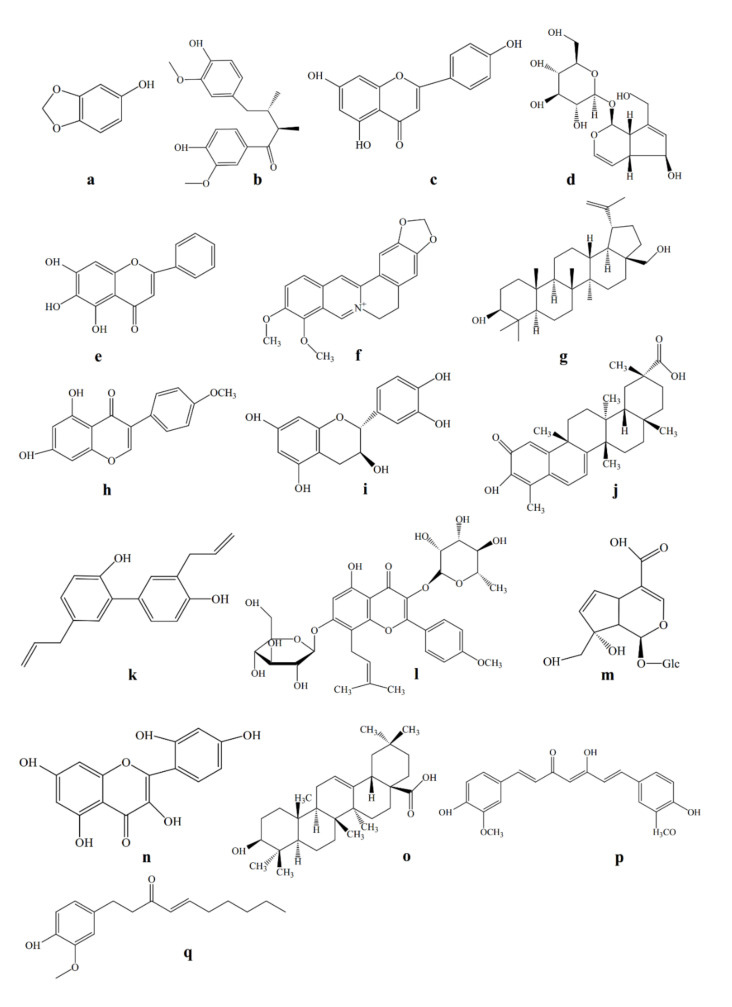
Chemical structure of chondroprotective natural compounds—(**a**) sesamol, (**b**) cinnamophilin, (**c**) apigenin, (**d**) acubin, (**e**) baicalein, (**f**) berberine, (**g**) botulin, (**h**) biochanin A, (**i**) catechin, (**j**) celastrol, (**k**) honokial, (**l**) icarin, (**m**) monotropein, (**n**) morin, (**o**) oleanic acid, (**p**) curcumin, and (**q**) 6-shogaol.

The actions of pro-inflammatory cytokines were found to be blocked by chondroprotective substances. Recently, natural herbs used in the form of traditional medicine have led to a restoration of scientific interest in their biological effects. Application of traditional medicinal plants for the treatment of OA has become attractive as they are claimed to show clinical efficacy with minimal side effects. Additionally, medicinal plants are frequently inexpensive, locally available, and easily consumable. Numerous inhibitors of the MMPs were proposed as potential therapeutic agents, the various types of compounds (as shown Figure 1) and their activities were reviewed [60], as follows:

### 7.1. Sesamol

**Figure 2:**
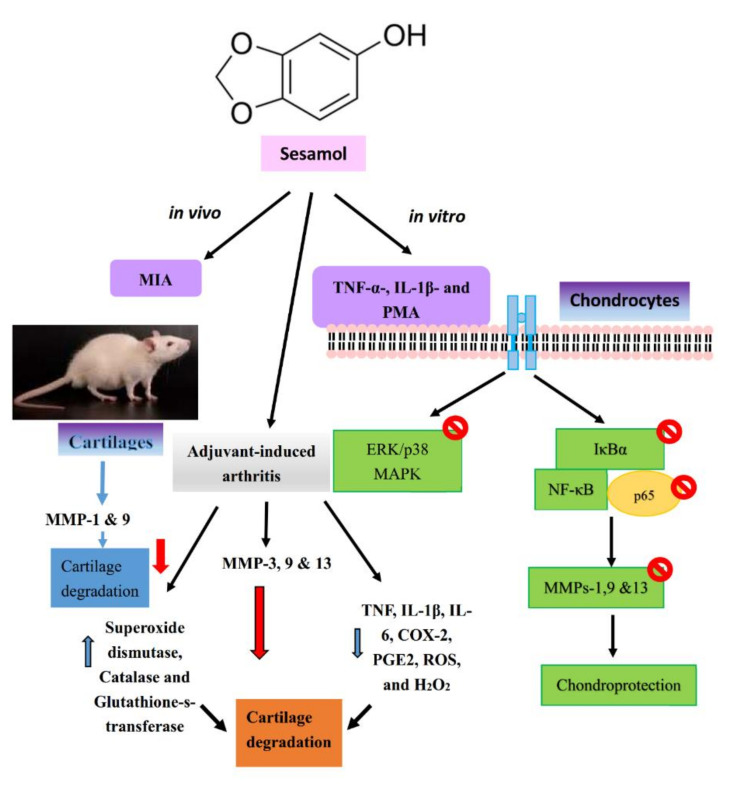
In vitro and in vivo chondroprotective molecular targets of sesamol.

In India and other East Asian countries, sesame (*Sesamum indicum*) has been taken as a habitual health food [61], its oil was found to be effective for the cure of various diseases, including atherosclerosis, hypertension, and aging [62]. Phenol, sesamin, sesamol, sesamolin, and a little amount of tocopherol are the major constituents of sesame oil that contribute to its oxidative strength [63]. Sesamol is a key compound of sesame oil, which is more tolerable against oxidative damage than other plant oils [64]. The anti-MMP-9 [65], anti-inflammatory [66], anti-oxidant [67], anti-injury, and wound healing [68] properties of sesamol, reveal its potential therapeutic value. Our previous study found that sesamol attenuated MMPs-1, -9, and -13 expression in TNF-α-, IL-1β-, or PMA-stimulated chondrocytes, through the inhibition of NF-κB and ERK/p38 MAPK [37], as shown in Figure 2. This study also found that sesamol expressively inhibited MMPs expression on the cartilage of MIA-induced OA rats [37]. A study found that animal model of MIA-induced OA, mimicked human OA in terms of inflammatory response and cartilage degeneration [69]. Moreover, MIA induced OA in animals could be useful to study the efficiency and mechanism of therapeutic agents against OA [70].

Sesamol was confirmed to recover arthritis-induced cartilage degeneration, by alleviating increased serum levels of hyaluronidase and MMPs-3, -9, and -13 [71]. This compound was also found to be protective in bone resorption, by decreasing bone joint exoglycosidases, cathepsin D, and tartarate-resistant acid phosphatases. It also abolished the inflammatory markers of TNF, IL-1β, IL-6, COX-2, prostaglandin E2 (PGE2), reactive oxygen species (ROS), and hydrogen peroxide (H_2_O_2)_ [71]. Moreover, sesamol was found to counteract arthritis-induced oxidative stress, by repairing the antioxidant defense systems, by elevating the activities of superoxide dismutase, catalase, and glutathione-s-transferase, and reducing ROS [71]. These outcomes can offer novel molecular insight for the development of natural therapeutics on destructive joint diseases.

### 7.2. Cinnamophilin

**Figure 3:**
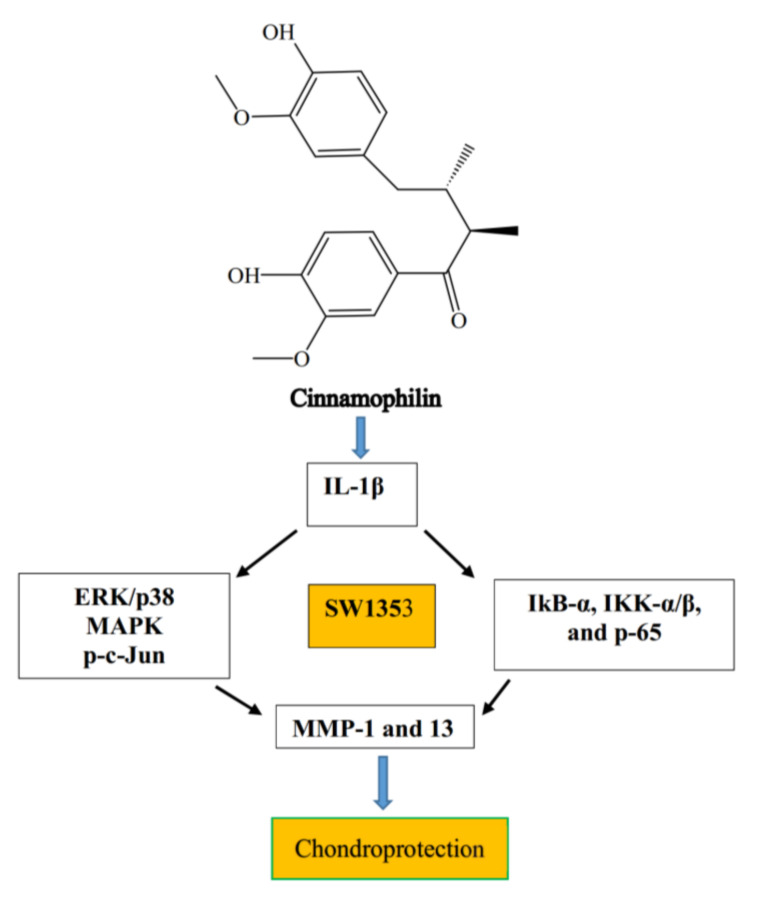
Chondroprotective mechanisms of cinnamophilin.

Cinnamophilin was isolated from *Cinnamomum philippinense* and found to be a novel antioxidant and free radical-scavenging agent [72]. This compound was highly lipid soluble and thus had the potential to cross the blood–brain barrier (BBB) to the brain. It served as thromboxane synthase and the thromboxane A_2_ receptor [73], and block Na^+^ and Ca^2+^ inward currents in rat cardiac cells [74]. Cinnamophilin was demonstrated to protect ischemic-reperfusion injury [75], and reduce brain infarction and transient focal cerebral ischemia in mice [76,77]. Our earlier study also established that cinnamophilin protects cells against oxidative stress and inhibits oxidative modification of human low-density lipoprotein (LDL) [72]. Another study from our group found that cinnamophilin inhibits IL-1β-induced expression of MMPs in human SW1353 chondrosarcoma cells [78]; cinnamophilin at concentrations of 5, 10, 20, and 30 mM, significantly inhibited IL-1β induced expressions of MMP-1 and 13. In addition, we found that cinnamophilin reduced the IL-1β-induced phosphorylation of IKK-α/β and degradation of IκB-α. The p-p65 levels in the chondrocytes of cinnamophilin-treated cells decreased markedly, compared to those in cells treated with IL-1β alone [78]. These results indicated that cinnamophilin might act as chondroprotective agent by mitigating collagen matrix breakdown in the cartilage of damaged tissues, such as those found in arthritic disorders (Figure 3).

### 7.3. Apigenin

Apigenin, bioactive, and anti-inflammatory flavonoid components in plants, received increased interest, due to their strong anti-inflammatory activities and their prospective roles in the prevention of cancers [79]. In primary cultured rabbit chondrocytes, apigenin found to inhibit the gene expression of *MMP-1, 3,* and *13* [80] and also diminished the proteolytic activity and secretion of MMP-3. Additionally, apigenin repressed MMP-3 in rat knee joint [80]. Administration of apigenin (40 and 20 mg/kg) was reported to suppress the phosphorylation of NF-κB p65, IκB kinase α (IKKα), iκB kinase β (IKKβ), and IκB-α in adjuvant-induced arthritis rats, which recommended that the inhibition of NF-κB activation by apigenin might be due to the inhibition of the NF-κB signaling pathways [81]. Apigenin is reported to relieve pain and paw swelling, and mitigate inflammatory responses in adjuvant-induced arthritis; these findings might support that apigenin could be served as a novel therapeutic drug for treating arthritis.

### 7.4. Aucubin

Aucubin is a natural anti-inflammatory iridoid glucoside derived from various plants, including leaves of *Aucuba japonica* and *Eucommia ulmoides* [82], which is known for several pharmacological effects [83]. Aucubin was reported to inhibit inflammatory response by hindering the phosphorylation and degradation of IκB, and the translocation of NF-κB p65 in IL-1β-induced rat articular chondrocytes [84]. Moreover, this compound reduced the production of nitric oxide (NO) and the expression of induced nitric oxide synthase (iNOS), cyclooxygenase-2 (COX-2), and MMPs in induced chondrocytes [84]. Chondrocytes treated with aucubin reported to show a significant ROS scavenging effect and also inhibited H_2_O_2_-induced apoptosis and necrosis in chondrocytes by reducing caspase-3 activity [85]. 

### 7.5. Baicalein

Baicalein, a natural product derived from the roots of *Scutellaria baicalensis*, is branded as Huang Qin in Chinese traditional medicine [86]. Baicalein is paid attention to in the field of pharmaceutical, cosmetic, and food industry, due to its excellent biological action. This compound was described to inhibit the expression of MMP-3 and 13 in human chondrocytes [87]. A previous study found that baicalein inhibited IL-1β-induced expression of caspase-3 in chondrocytes and stimulated B cell lymphoma 2 (Bcl-2) expression. In chondrocytes, IL-1β induced expression of COX-2, MMP-3, and -9 were suppressed by baicalein [88]. Baicalein and baicalin exhibited therapeutic effects against arthritis and colitis. These two compounds were reported to control the activation of target cells in these autoimmune diseases, via the signal transducer and activator of transcription (STAT) subtypes in the janus kinase (JAK)-STAT pathway [89].

### 7.6. Berberine

Berberine, an anti-inflammatory natural compound extracted from *Rhizoma coptidis*, was described to inhibit cartilage degradation and to suppress the NF-κB signaling pathways, in a human chondrosarcoma cells. Additionally, a strong chondroprotective effect was found in berberine treated chondrocytes by inhibition apoptosis and MMP-1, -3, and -13 protein and gene expressions [90,91,92,93]. The inhibitory effects of berberine on RA was associated with an increase in cyclin-dependent kinase (CDK) inhibitors Cip1/p21 and Kip1/p27 and a reduction of CDK2, CDK4, and CDK6, and cyclins D1, D2, and E [94]. Additionally, berberine was found to augment apoptotic death in RA, which was found to be connected with an intensified expression of Bax, reduced expression of Bcl-2 and Bcl-xl, interruption of mitochondrial membrane potential, and stimulation of caspase-3, caspase-9, and poly polymerase [94].

### 7.7. Betulin and Biochanin

Betulin, a natural anti-inflammatory compound dereived from *Betulae cortex*, was reported to inhibit IL-1β-induced gene expression of MMP-1, 3, and 13. Betulin blocked in vivo MMP-3 production in rat knee joint [95]. Biochanin A, an isoflavone derived from red clover, showed pontential anticancer, antiallergic, and anti-inflammatory effects [96]. A previous study found biochanin A had anticancer property against human glioblastoma (U87MG) cells, through inhibition of the enzymatic activities of MMP-2 and -9 [97]. Considering the fact that MMPs are considered to be major factors in the progression of OA, this close correlation between biochanin A and MMPs, inspired the researchers to discover if biochanin A might have a protective effect in OA through the regulation of MMPs. As expected, this compound blocked protein and mRNA expression of MMPs-1, -3, and -13, and induced tissue inhibitors of metalloproteinase (TIMP-1) expression in IL-1β induced chondrocytes, by regulating the NF-κB signaling pathway [98].

### 7.8. Catechin

Catechin is a major compound of *Chaenomeles speciosa* (sweet) Nakai [99], green tea [100], *Acacia catechu* (L.f.) wild [101], and *Polygonum bistorta* [102]. Earlier studies showed that green tea hampered arthritis progress in a mouse model [103], hindered the proteoglycan breakdown and release from OA and RA cartilage treated with IL-1β and TNF-α of human cartilage [104]. Another study found that Epigallocatechin-3-gallate (EGCG) inhibited IL-1β-induced cartilage proteoglycan degradation, and MMP-1 and 13 release and expression in human chondrocytes [105]. This effect might be attributed via the inhibition of NF-κB and activator protein (AP-1) activation, and its promoter binding activity [105]. In chondrocytes, the gene expression of MMP-13 is strongly regulated by NF-κB and AP-1/c-Jun, and therefore inhibition of these transcription factors could result in the attenuation of MMP-13 [18]. Previous studies also showed that EGCG inhibited the IL-1β-induced elevation of NF-κB and AP-1/c-Jun in human chondrocytes, by blocking their nuclear translocation and activation [106,107,108]. Catechin, was reported to show chondroprotective activity by hindering IL-1β-stimulated expression of IL-8, PGE2, and COX-2, in human synovial fibroblasts [109]. An interesting study found reduced levels of MMP-1, -3, -8, -13, a disintegrin and metalloproteinase with thrombospondin motifs (ADAMTS5), IL-1β, and TNF-α mRNA, and elevated gene expression of the MMP regulator Cbp/p300 interacting transactivator 2 (CITED2) in mice of EGCG-treated articular cartilages [110]. These groups also found that mice treated with EGCG showed reduced OA-associated pain, as evidenced by higher locomotor behavior [110].

### 7.9. Celastrol, Crocin, and Ginsenosides

Celastrol, also called tripterine, the richest and most gifted bioactive compound derived from the root of the *Tripterygium wilfordii* plant, is used in Chinese medicine to treat several immunological disorders, including RA. This compound displayed a protective effect via the inhibition of IL-1β-induced protein and mRNA expression of MMP-13, -3, and -1, and COX-2, and iNOS-2 in primary human osteoarthritic chondrocytes, without inducing cytotoxicity [111]. The same authors found that stimulation of chondrocytes with IL-1β caused dramatic luciferase activity, and this luciferase activity was expressively downregulated in celastrol-pretreated chondrocytes, which proposed that reduction of MMPs, iNOS, and COX-2 expression could be due to the inhibition of the NF-κB pathways. 

*Crocus sativus* L. is paid much attention due to its several beneficial properties, of which crocin is one of the most prevailing components. Liu et al. found that crocin decreased arthritis scores, paw swelling, and weight loss in type II collagen-induced arthritis (CIA) rats [112]. Additionally, crocin was found to reduce chondrocyte death, cartilage surface erosion, and bone erosion in CIA rats [112]. These authors also found that daily treatment of CIA rats with crocin significantly decreased the serum levels of MMP-1, -3, and -13 [112]. They also discovered that crocin decreased the serum levels of TNF-α, IL-17, IL-6, and CXCL8 in CIA rats [112]. Crocin was found to inhibit the expression of MMP-3, -11, and -13, via the NF-κB signaling pathway, in articular chondrocytes and reduce in vivo cartilage degeneration [113]. A natural product found in *Angelica sinensis*, namely ferulic acid, act as anti-osteoarthritic agent, through its blocking effect on H_2_O_2_-induced mRNA expression of MMP-1, -31, TNF-α, and IL-1β in chondrocytes [114]. Ginsenosides, derived from *Panax ginseng* presented various biological effects. Ginsenoside Rb1, a derivative of ginsenosides, was found to inhibit the levels of MMP-1, MMP-31, NO, iNOS, IL-1β, and TNF-α, and stimulate the expression of type II collagen [115,116]. The chondroprotective effect of ginsenosides Rg1, Rg3, Rg5, Rk1, Rf, Rd, Rc, and F4 were reported by Huang et al. [117] and Lee et al. [118].

### 7.10. Honokiol, Icariin, and Luteolin

Honokiol, an active and small molecule polyphenolic compound isolated from *Magnolia officinalis* was reported to recover IL-1β-stimulated expression of MMP-13, IL-6, iNOS, NO, COX-2, and PGE2 via the modulation of the NF-κB signaling pathway [119]. This compound potently inhibited collagen-induced arthritis, by reducing the production of cytokines, the expression of MMPs3, 9, and 13 and increasing antioxidant enzymes [120]. A previous study evaluated different passages of human-umbilical-cord-derived mesenchymal stem cells (hUC-MSCs), under honokila treatment, to examine the prime cell passage for chondrogenesis [121]. The results showed that the markers of SRY-related high-mobility group box 9 (SOX-9), aggrecan, and col2α1 were highly expressed in the second passage cells, which indicated that honokial enhanced chondrogenesis [121]. Icariin, a natural compound derived from *Epimedium pubescens*, hindered IL-1β-stimulated expression of MMP-13, and increased ECM synthesis to show its chondroprotective role [122]. Similarly, a flavonoid compound luteolin isolated from *Lonicerae flos* was found to inhibit IL-1β-stimulated gene expression, secretion, and enzyme activity of MMP-3, in articular chondrocytes [123]. This compound also repressed gene expression of MMP-1, -31, and ADAMTS-5, and recovered the production of MMP-3 protein in the rat knee joint [123]. 

### 7.11. Monotropein

Monotropein, a compound present in *Morinda officinalis*, was reported to recover IL-1β-stimulated expression of MMP-3 and 13 in chondrocytes [124]. A recent study found that monotropein attenuated TNF-α tempted iNOS, COX-2 MMP-1, MMP-3, and MMP-13 in chondrocytes. Further, this compound blocked TNF-α induced MAPK/NF-κB activation in chondrocytes [125]. The anti-apoptotic and anti-catabolic effects of monotropein was investigated in IL-1β-induced rat osteoarthritic chondrocytes. Altogether, these results proposed that monotropein showed anti-apoptosis and anti-catabolic activity in chondrocytes, which might sustain its possible therapeutic role in OA [124].

### 7.12. Morin

Morin, a dietary bioflavonoid found in guava, onion, apples, and the *Moraceae* group, are used as dietary suppelements and herbal medicines [126]. This compound showed antioxidant, cytoprotection, antimutagenesis, antidiabetic, and anticarcinogenic effects [127]. Morin was found to inhibit IL-1β in colitis in rats and block the production of NO and PGE2 in LPS-induced RAW 264.7 cells [128,129]. Morin was also reported to inhibit the production of NO and PGE2 and suppress iNOS and COX-2 in IL-1β induced in vitro chondrocytes, as well as in vivo OA models [130]. A combined morin and indomethacin treatment in arthritic rats found noteworthy reduction in articular elastase activity than morin-alone treated rats [131]. This study also investigated the effect of combined morin and indomethacin on cartilage and bone degradation by estimating the activities/levels of lysosomal acid hydrolases, glycoproteins, and bone collagen in arthritis rats. The results found that the combination therapy of morin with indomethacin, expressively stopped the degradation of bone and cartilage by modulating the lysosomal acid hydrolases, glycoproteins, bone collagen, and urinary constituents. This compound significantly reduced IL-1β-induced MMP-3 and -13 and increased TIMP-1 expressions via the destruction of extracellular regulated kinases (ERK1/2) and p38 phosphorylation in chondrocytes [132]. 

### 7.13. Oleanolic Acid

Oleanolic acid is a natural product derived from *Cornus officinalis*, which exhibited numerous biological activities, including anti-oxidative and anti-inflammatory properties [133], and it is reported to diminish renal ischemia/reperfusion injury by its antioxidant, anti-inflammatory, and anti-apoptotic properties [133]. In addition, it is reported to have a potent inhibitory effect against RA by controlling T cell immune responses [134], where it reduced Th1/Th17 phenotype CD4+ T lymphocyte growths and inflammatory cytokine productions in T cell activated draining lymph nodes and spleen. This compound also reduced the expression and production of cytokines and MMP-1 and 3 in the ankle joint tissue and RA synovial fibroblasts, via protein kinase B (Akt), mitogen activated protein kinases (MAPKs), and NF-κB signaling [134]. Moreover, oleanolic acid was found to stimulate the gene expression of type II collagen and blocked the gene expression of ADAMTS-4 and 5, MMP-1, and MMP-13, and the protein expression of MMP-3. Additionally, an in vitro enzyme activity and in vivo MMP-3 production found in oleanolic acid treated osteoarthritic chondrocytes [135].

### 7.14. Curcumin and Shogaol

Curcumin is a major curcuminoid natural product of turmeric and it was reported to have effective biological properties, including anti-inflammatory, antioxidant, and anticancer. This compound was exposed to have chondroprotective potential by alleviating OA disease pathogenesis and symptoms. A study found that curcumin exerted its chondroprotective effects by regulating MMP-13 and aggrecanase ADAMTS5 in chondrocytes [136]. Onodera et al. found that curcumin inhibited the macrophage migration inhibitory factor (MIF)-induced upregulations of MMP-1 and MMP-3 mRNAs, in cultured synovial fibroblasts of RA patients [137]. Henrotin et al. revealed the molecular targets of curcumin via the gene expression AP-1 and NF-κB signaling in chondrocytes [138]. These authors proved the hypothesis in which curcumin protect human chondrocytes from the IL-1β-induced cellular and morphological changes. In addition, they investigated the anti-apoptotic effects of curcumin in IL-1β-stimulated human chondrocytes. Curcumin acted as an inhibitor of AP-1 to inhibit the actions of osteosarcoma cells and it was evidenced on the activation of the MEK/ERK and activator of STAT pathways in oncostatin M (OSM) signaling [139]. A well-arranged study demonstrated that the treatment of chondrocytes with curcumin blocked IL-1β and TNF-αinduced NF-κB activation, via the inhibition of IκBα phosphorylation, IκBα degradation, p65 phosphorylation and p65 nuclear translocation. These measures were associated with downregulation of the NF-κB targets, including COX-2 and MMP-9 [55].

Ginger (*Zingiber officinale*) is a traditional Asian medicine and it is used to treat a variety of rheumatic diseases, including OA [140,141]. Bioactive compounds in ginger rhizome extract, namely gingerols and shogaols [142] were found to have anti-inflammatory properties. Compared to gingerols, 6-shogaol had the maximum anti-inflammatory activity in innate immune cells, which was evidenced from both in vivo and in vitro experiments [143,144]. 6-shogaol inhibited LPS–stimulated MMPs-2 and 9 induction in chondrocytes [145]. A study found that 6-shogaol was effective in reducing the swelling of complete Freund’s Adjuvant (CFA)-induced monoarthritis rat knees. This effect was found to be associated with reduced levels of soluble vascular cell adhesion molecule-1 (VCAM-1) in the blood and infiltration of leukocytes, including lymphocytes and monocytes/macrophages, into the synovial cavity of the knee [144] (Table 1). These authors also observed the protection of the morphological integrity of the cartilage lining in the femur.

**Table 1:** Molecular targets of natural compounds on chondroprotection.

S. No	Compounds Name	OA/RA Stimulators	Molecular Targets	References
1	Sesamol	TNF-α-, IL-1β- or PMA in SW1353 cells	Reduced MMPs-1, -9, and -13 expression, MAPKs expression and NF-κB signaling pathway	[37]
		MIA in rats	Reduced MMPs-3, -9, and -13 and bone joint exoglycosidases, cathepsin D and tartarate-resistant acid phosphatases	
		Adjuvant-induced arthritis	Reduced TNF, IL-1β, IL-6, COX-2, PGE2, ROS, and H_2_O_2_	
2	Cinnamophilin	IL-1β in SW1353 cells	Reduced MMPs-1 and 13 expression	[78]
			Decreased IKK-α/β and degradation of IκB-α and p-p65 expression	
3	Apigenin	IL-1β in rabbit chondrocytes and rat knee	Decreased MMP-1, -3, and -13 expression	[80]
		Adjuvant-induced arthritis rats	Reduced MMP-3 expression	[81]
			Reduced NF-κB p65, IKK-α, IKK-β and IκB-α expression	
			Alleviated pain and paw swelling	
4	Aucubin	IL-1β in rat chondrocytes	Recovered NF-κB p65 and IκB-α	[84]
			Reduced NO production and iNOS, COX-2 and MMPs expression	
			Increased ROS scavenging	
		Mechanical stimulus	Decreased apoptosis and necrosis	[85]
		H_2_O_2_	Reduced caspase-3 expression	
5	Baicalein	IL-1β in chondrocytes	Decreased caspase-3, COX-2, MMPs-3 and -9 expression	[88]
			Increased Bcl-2 activation	
		Arthritis and colitis	Regulates JAK-STAT pathway	[89]
6	Berberine	IL-1β in chondrocytes	Decreased MMPs via the Akt pathway	[91]
			Decreased IL-1β and cartilage degradation	
		CCN2	Increased CDK inhibitors Cip1/p21 and Kip1/p27; Decreased CDK2, CDK4, and CDK6, and cyclins D1, D2 and E;	[92]
			Reduced caspase-3 and -9	[94]
7	Betulin	IL-1β in chondrocytes	Decreased MMPs-1, -3, and -13 expression	[95]
		Rat knee joint	Increased type-II collagen gene expression	
			Decreased MMP-3 expression	
8	Biochanin	IL-1β in chondrocytes	Decreased mRNA and protein of MMPs-1, -3, and -13	[98]
			Increased TIMP-1 mRNA and its protein	
			Decreased IκB-α degradation and NF-κB activation	
9	Green tea	IL-1β and TNF-α	Decreased proteoglycan breakdown and release from OA and RA cartilage	[104]
10	EGCG	IL-1β in chondrocytes	Decreased cartilage proteoglycan degradation, and MMPs-1 and -13 release and expression	[105]
			Decreased the activation and promoter binding activity of NF-κB and AP-1	
			Decreased MMP-13, NF-κB, AP-1/c-Jun, and p38	
		IL-1β in chondrocytes	Decreased MMPs-1, -3, -8, -13, ADAMTS5, IL-1β, and TNF-α mRNAs	[106,107,108]
		Articular cartilages	Increased CITED2 and decreased OA pain	[110]
11	Catechin	IL-1β in chondrocytes	Decreased IL-8, PGE2, and COX-2	[109]
12	Celastrol	IL-1β in chondrocytes	Decreased protein and mRNA expression of MMPs-1, -3, -13, COX-2, and iNOS-2	[111]
			Decreased NF-κB pathways	
13	Crocin	Type II collagen-induced arthritis in rats	Decreased arthritis scores, paw swelling, and weight loss	[112]
			Decreased chondrocyte death, cartilage surface erosion, and bone erosion	
			Decreased MMPs-1, -3, and -13 expression	
		IL-1β	Decreased TNF-α, IL-6, IL-17, and CXCL8	[113]
		Rabbit cartilages	Decreased NF-κB pathways	
			Decreased degeneration of cartilage	
14	Ferulic acid	H_2_O_2_	Decreased mRNA expression of MMPs-1, -13, TNF-α, and IL-1β	[114]
15	Ginsenosides	H_2_O_2_ and	Decreased MMPs-1, -13, NO, iNOS, IL-1β, and TNF-α	[115,116]
		IL-1β	Increased type II collagen expression	
16	Honokial	IL-1β in chondrocytes	Decreased MMP-13, IL-6, iNOS, NO, COX-2, and PGE2	[119]
			Decreased NF-κB signaling pathway	
		Type II collagen-induced arthritis in rats	Decreased MDA, IL-1β, and TNF-α	[120]
			Increased GSH, CAT and SOD	
18	Icarin	IL-1β in chondrocytes	Decreased MMP-13 expression	[122]
			Increased extracellular matrix synthesis	
19	Luteolin	IL-1β in chondrocytes	Increased gene expression, secretion, and enzyme activity of MMP-3	[123]
			Increased gene expression of *MMP-13* and ADAMTS-5	
		Rat knee joint	Decreased MMP-3 expression	
20	Monotropein	IL-1β in chondrocytes	Decreased MMPs-3 and 13	[124]
		TNF-α in chondrocytes	Decreased iNOS, COX-2, MMP-1, -3, and -13	[125]
			Decreased MAPK/NF-κB	
21	Morin	IL-1β in chondrocytes	Decreased NO, PGE2, iNOS, and COX-2	[130]
			Inhibited degradation of bone and cartilage via regulation of the activities/levels of lysosomal acid hydrolases, glycoproteins, bone collagen, and urinary constituents	[132]
			Decreased MMPs-3 and 13, and TIMP-1	
			ERK1/2 and p38	
22	Oleanolic acid	Type II collagen-induced arthritis in rats	Decreased Th1/Th17 phenotype CD4+ T lymphocyte expansions	[134]
			Decreased expression and production of cytokines and MMPs-1 and 3	
			Decreased Akt, MAPKs, and NF-κB	
			Inhibited ADAMTS-5, MMPs-1, -13, and *ADAMTS-4* gene expression	
		Type II collagen-induced arthritis in rats	Decreased MMP-3 protein expression	[135]
			Inhibited in vitro enzyme activity and in vivo MMP-3 production	
23	Curcumin	DMM induced OA in mice	Decreased proteoglycan loss, cartilage erosion, synovitis and subchondral plate thickness	[136]
			Decreased IL-1β and TNF-α, MMPs -1, 3, and 13, and aggrecanase ADAMTS5	[137]
		MIF induced synovial fibroblasts of RA patients	Decreased MMPs-1 and -3 mRNAs	[138]
		IL-1β-induced chondrocytes	Recovered cellular and morphological changes	
		IL-1β and TNF-α induced chondrocytes	Decreased caspase-3 via AP-1 and NF-κB	[139]
			Decreased COX-2, MMP-9	
			Decreased NF-κB, IκB-α phosphorylation, IκB-α degradation, p65 phosphorylation, and p65 nuclear translocation	
24	6-Shogaols	CFA-induced monoarthritis in rats	Decreased paw edema via VCAM-1	[144]
		LPS-stimulated chondrocytes	Decreased MMPs- 2 and 9 induction	[145]

Abbreviations: MMP—Matrix metalloproteinases; TIMP—Tissue inhibitors of metalloproteinases; ADAMTS—A Disintegrin and metalloproteinase with thrombospondin motifs; iNOS—Inducible nitric oxide synthase; COX-2—Cyclooxygenase-2; PGE2—Prostaglandin E2; MAPKs—Mitogen-activated protein kinases; NF-κB—Nuclear factor-κB; GSH—Reduced glutathione; CAT—Catalase; SOD—Superoxide dismutase; MDA—Malondialdehyde; IL-1β—Interleukin-1β; TNF-α—Tumor necrosis factor-α; NO—itric oxide; JAK—Janus kinase; STAT—Signal transducer and activator of transcription; CDK—Cyclin-dependent kinase; H_2_O_2_—Hydrogen peroxide; ROS—Reactive oxygen species; AP-1—Activator protein 1; MIA—Monosodium iodoacetate; PMA—Phorbol 12-myristate 13-acetate; DMM—Destabilization of the medial meniscus; MIF—Macrophage migration inhibitory factor; LPS—lipopolysaccharides; and CFA—Complete freund’s adjuvant.

## 8. Conclusions and Future Direction on Therapy for Osteoarthritis

The non-steroidal anti-inflammatory drugs are the most commonly recommended drugs to treat arthritis patients, but they still have unwanted side effects. Due to these limitations, most arthritis patients have started trying natural products/traditional Chinese medicine to release symptoms and related illnesses. Natural products are extensively exposed for treatment of different diseases, such as cancer, infectious, and autoimmunity diseases. Conversely, there is no sufficient information about their mechanism of action on the protective role against the destructive joint diseases, and thus, describing the mechanism of action of natural products is a warrant investigation. In this review, substantial authentication of various natural products and their mechanism of action for the treatment of arthritis is summarized.

A conservative controlling of osteoarthritis cannot discourse the major cause of the disease, when the application of agents is used alone. Additionally, these agents are not acceptable for long-term control of osteoarthritis, as they display major side effects. In contrast, varieties of natural products show protective effects against proinflammatory cytokine-induced expression and the catabolic activity of MMPs in articular cartilage, via the regulation of the NF-κB signaling pathway. Natural products exhibited inhibitive effects on the apoptosis in chondrocytes, and decline in the production of the ECM in articular cartilage. Nevertheless, although several preclinical and clinical studies are directed so far in natural product chemistry, still there are no perfect natural products recommended as an antagonist to the progression of the symptoms of osteoarthritis. This review might provide absolute readings about how natural compounds are beneficial for the treatments of joint diseases. Additionally, the information of the chondroprotective mechanism of natural substances would afford new opportunities to promote therapeutic strategies projected at encouraging destructive joint disorders.
